# Secondary Organizing Pneumonia: A Case Report of a Noteworthy Complication of Breast Irradiation

**DOI:** 10.7759/cureus.78374

**Published:** 2025-02-02

**Authors:** Teresa B Rosa, Catarina Novalio, Isabel Duarte, Dina Matias, Teresa Guiomar

**Affiliations:** 1 Radiation Oncology, Instituto Português de Oncologia de Lisboa Francisco Gentil, Lisbon, PRT; 2 Radiology, Instituto Português de Oncologia de Lisboa Francisco Gentil, Lisbon, PRT; 3 Pulmonary Oncology, Instituto Português de Oncologia de Lisboa Francisco Gentil, Lisbon, PRT

**Keywords:** breast cancer, breast cancer radiation, radiation-induced organizing pneumonia, radiation therapy, secondary organizing pneumonia

## Abstract

Organizing pneumonia (OP) is a pattern of pulmonary tissue repair injury. It can be cryptogenic or a response to a specific lung injury. Radiation therapy is a potential cause of secondary OP, especially after breast irradiation. The lesions may appear anywhere in the lung parenchyma with no relation to the irradiated side. Corticotherapy is the mainstay of treatment and prognosis is generally favorable. We describe a case of a female patient previously diagnosed with breast cancer who developed secondary OP within six months of postoperative radiotherapy. Initial diagnosis was challenging due to the rarity of the disease as well as management that required close follow-up with careful tapering given the frequent relapses associated with the withdrawal of corticosteroid therapy. The reported case emphasizes the importance of recognizing secondary OP as a rare complication of postoperative radiotherapy for breast cancer and puts into view the complexity of its management.

## Introduction

Organizing pneumonia (OP) is a pattern of pulmonary tissue repair after injury. It can be cryptogenic or a response to a specific lung injury and is also observed histopathologically in many diverse clinical contexts. Cryptogenic organizing pneumonia (COP) has no identifiable cause and is classified as a form of idiopathic interstitial pneumonia. Secondary forms of OP are attributable to a specific cause (e.g., viral infection, drug toxicity, inhalation injury, radiation therapy, and cancer) [1].

The pathophysiologic mechanism has not yet been clearly described but it is thought to be due to alveolar injury, leading to the formation of organized granulation tissue that obstructs the alveoli and bronchioles; obstructions can result in progressive respiratory failure if left untreated [2].

OP has been reported worldwide; however, the exact incidence and prevalence are unknown. Two Japanese epidemiological studies from 2015 and 2018, showed an incidence of postradiotherapy OP in breast cancer patients of 1.4% while a meta-analysis from 2020 reported an incidence of 1.0% to 3.0% [3-5].

## Case presentation

A 73-year-old woman with a medical history of tuberculosis at age 12 with no sequelae and allergies to beta-lactams, aspirin, nitrofurantoin, and sulphonamides was diagnosed with an early staged breast cancer on the left side, and underwent lumpectomy, without any postoperative complications. The pathology report revealed a 2.5 x 20 x 2.5 mm invasive ductal carcinoma of grade 2, with estrogen receptors at 50%, progesterone receptors at 80%, and HER-2 and sentinel node-negative (pT1b pN0(sn) M0). At a multidisciplinary meeting, adjuvant radiotherapy and hormonal therapy for five years (tamoxifen 20 mg id) were proposed.

The prescribed radiotherapy dose was 50 Gy to the left breast with a sequential 10 Gy boost to the tumor bed (tangential irradiation) and the treatment was well tolerated with only grade 1 radiation dermatitis.

Six months after the end of radiation therapy, the patient was admitted to the ER with a two-week-long history of fatigue and dyspnea on exertion, without a fever, cough, or sputum. The patient had no history of smoking or substance abuse, no history of asthma, bronchitis, rheumatologic diseases, connective tissue disorders, or inflammatory bowel disease, and no record of ambiental or occupational exposures or ATM mutations [6].

**Figure 1:**
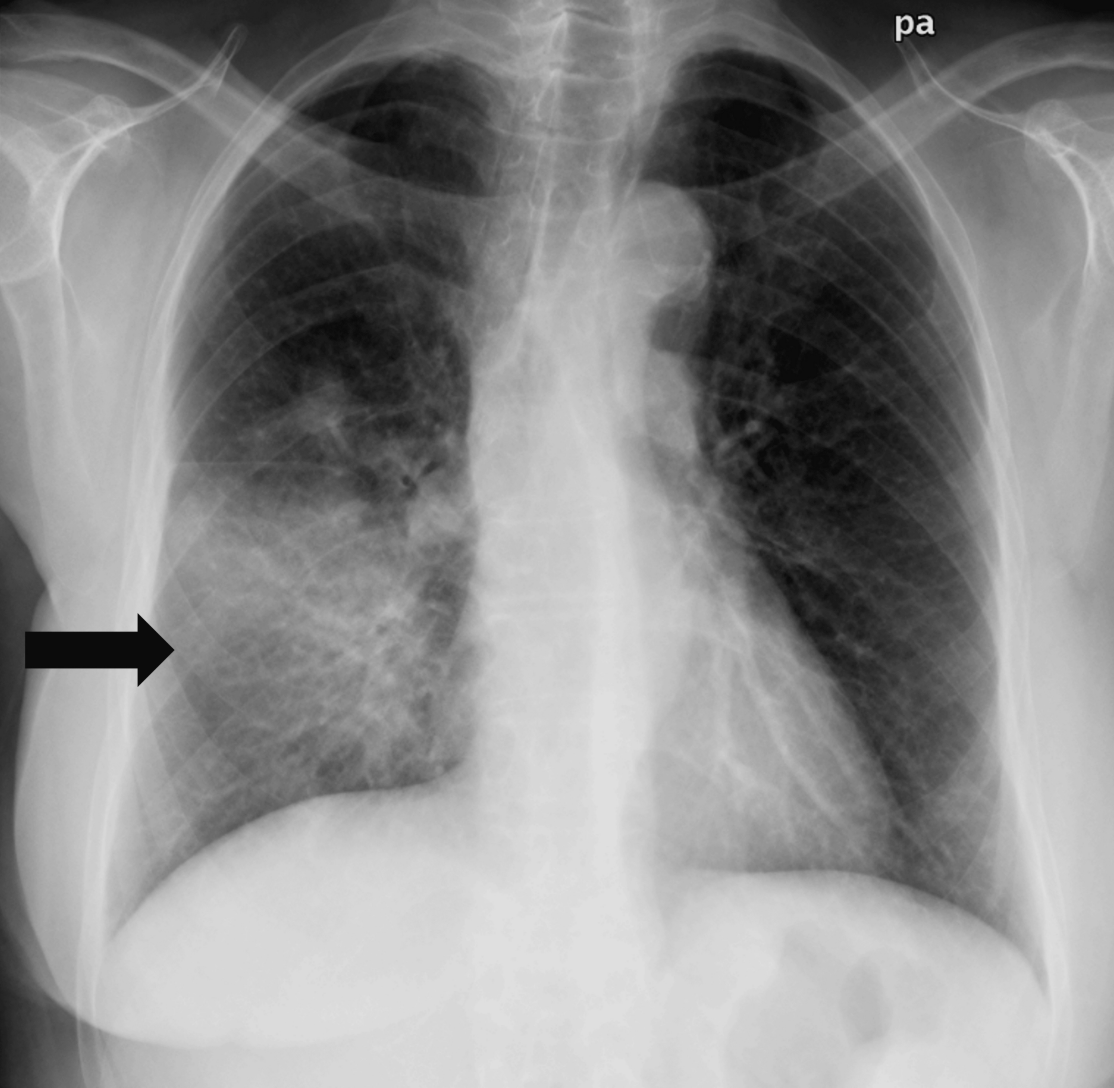
Initial thoracic X-ray showing right lung opacities (black arrow).

Pulmonary rales were present at auscultation of the right hemithorax, and a thoracic X-ray (Figure 1) revealed right lung opacities.

Blood tests revealed minor neutrophilia with an absolute neutrophil count of 7210 and a C-reactive protein value of 10.18 mg/dL. Peripheral oxygen saturation was 97% and arterial blood gas analysis showed a partial pressure of oxygen (pO2) of 82 mmHg, a partial pressure of carbon dioxide (pCO2) of 35 mmHg, and a pH of 7.46 (Table 1). Blood cultures were taken, which came back negative.

**Table 1:** Patient's initial analytic parameter values and corresponding normal ranges. PO2: partial pressure of oxygen; PCO2: partial pressure of carbon dioxide.

Analytic parameters	Patient's values	Normal range
Neutrophils absolute count	7210 cells/μL	2500-7000 cells/μL
C-reactive protein	10.18 mg/dL	<0.5 mg/dL
Peripheral oxygen saturation	97%	95-100%
PO2 arterial blood	82 mmHg	75-100 mmHg
PCO2 arterial blood	35 mmHg	38-42 mmHg
pH arterial blood	7.46	7.38-7.42

**Figure 2:**
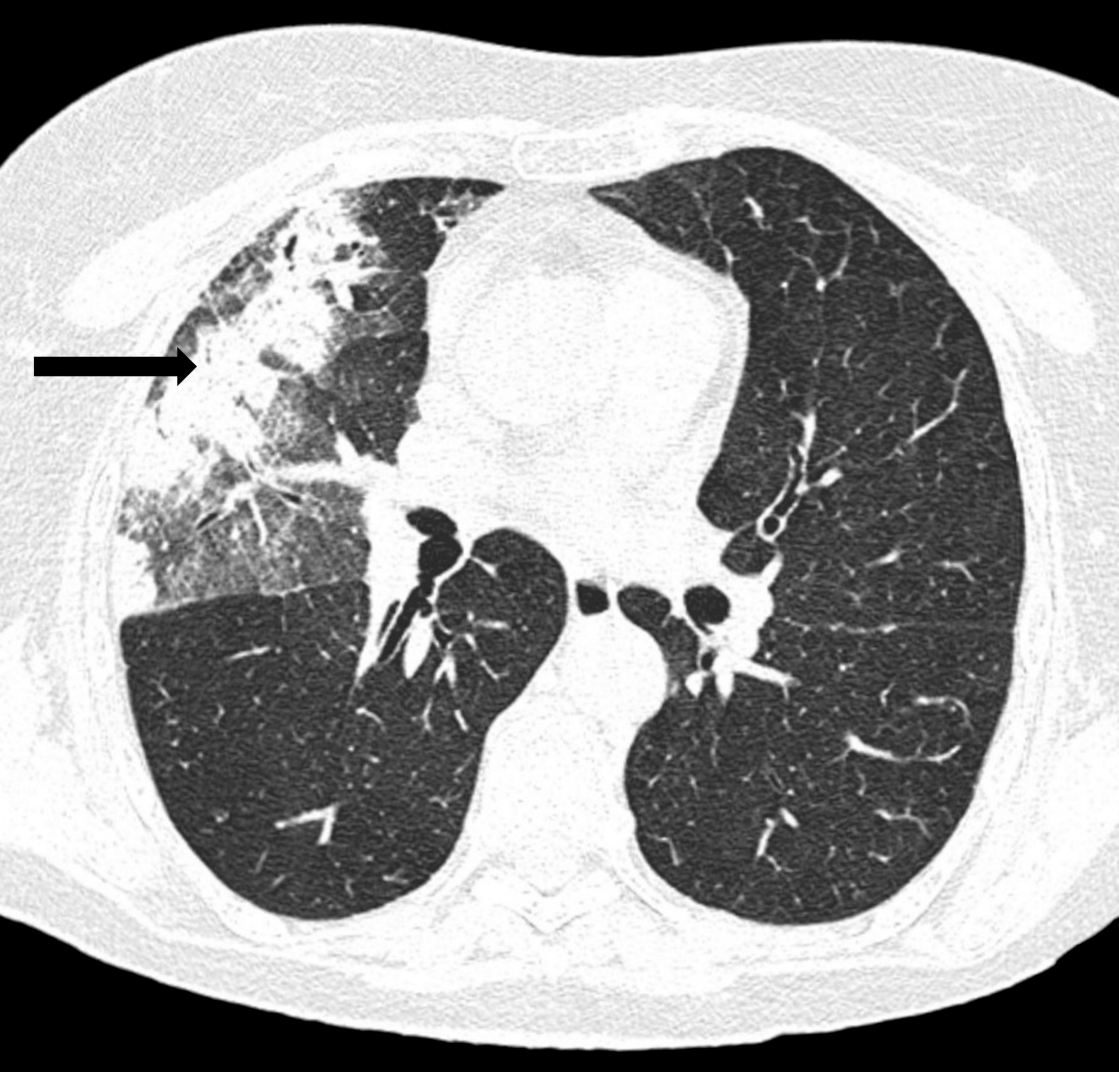
Thoracic CT scan revealing right parenchymal consolidation, permeated by air bronchograms. Parenchymal consolidation in the right lung, permeated by air bronchograms (black arrow).

The patient was first treated empirically as an outpatient with a course of fluoroquinolone antibiotics (ciprofloxacin 500 mg twice daily) but four days later developed pyrexia and her clinical status deteriorated. She was admitted to the hospital and a thoracic computed tomography (CT) scan revealed right lung consolidations suggestive of pneumonia (Figure 2).

A change to levofloxacin (750 mg once daily) was attempted but after 48 hours of continuous deterioration, it was shifted to a macrolide antibiotic (clarithromycin 250 mg twice a day) coupled with high-dose corticosteroid therapy (methylprednisolone 1 mg/kg/day), resulting in clinical and radiological improvement. The pulmonary function tests only showed a mild reduction of the diffusing capacity of the lungs for carbon monoxide (DLCO) (69.4%) and the patient was discharged. After 10 days, she remained well and began tapering corticosteroids.

**Figure 3:**
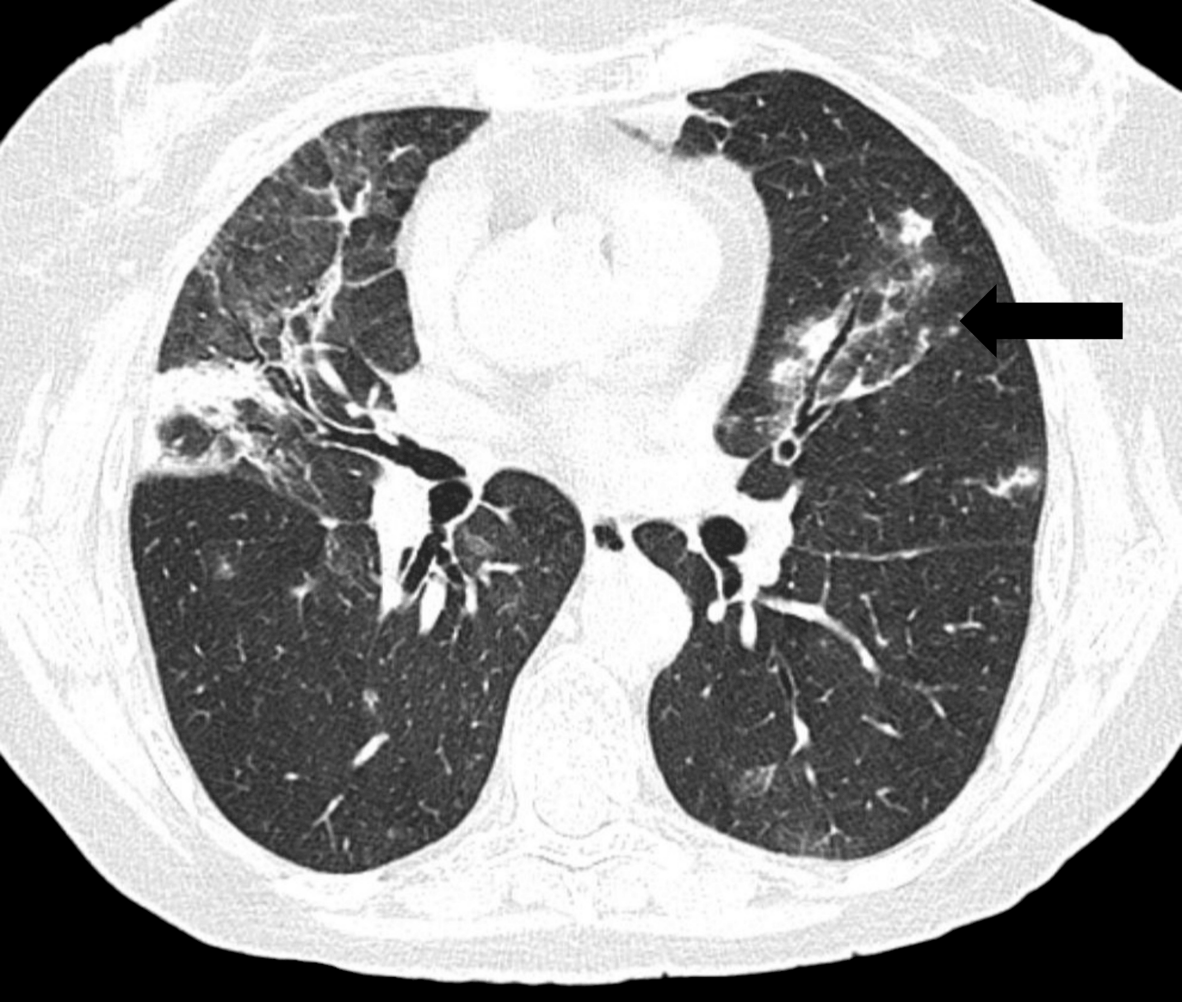
Thoracic CT scan showing new scattered ill-defined consolidation on the left lung despite reduction in right middle lobar parenchymal consolidation (first relapse). New parenchymal consolidations in the left lung (black arrow).

One month into the process of the tapering corticotherapy, the same set of symptoms reappeared accompanied by worsening noted on chest CT findings (Figure 3).

Additional investigation was performed, including bronchoscopy with bronchoalveolar lavage (BAL), which revealed increased cellularity at the expense of lymphocytes, and bronchial biopsy, which showed the presence of respiratory mucosa with chronic inflammation. BAL cultures were negative with no bacterial growth (Table 2).

**Table 2:** Diagnostic tests performed during the first episode of relapse and respective results.

Diagnostic tests performed	Results
Bronchoalveolar lavage (BAL)	Increased cellularity due to the increased number of lymphocytes present
Bronchial biopsy	Normal respiratory mucosa with chronic inflammatory changes
BAL cultures	Negative

Taking into consideration that the patient had a history of postoperative radiotherapy to the left breast in the six months that preceded the symptoms, a differential diagnosis was made with secondary cryptogenic pneumonia. The patient was prescribed prednisolone 30 mg/day, and one month after having restarted corticosteroid therapy, symptoms were resolved and slow dose tapering was initiated.

**Figure 4:**
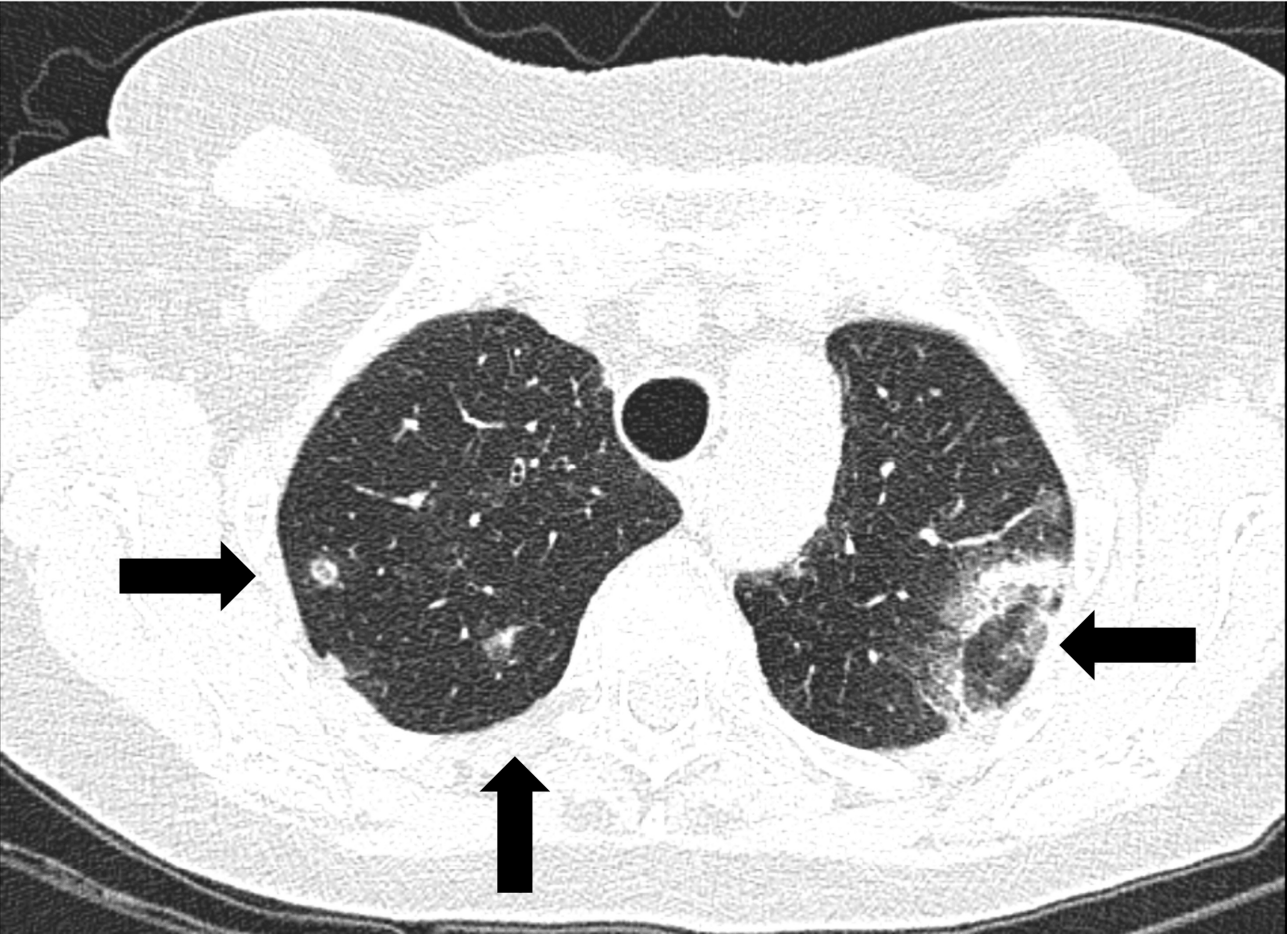
Thoracic CT scan revealing new areas of parenchymal consolidation localized to both pulmonary apexes (new location; second relapse). New areas of consolidation at the pulmonary apexes bilaterally (black arrows).

About two weeks later, the patient experienced extreme fatigue, and the disease relapsed with the appearance of new pulmonary migratory infiltrates (Figure 4).

**Figure 5:**
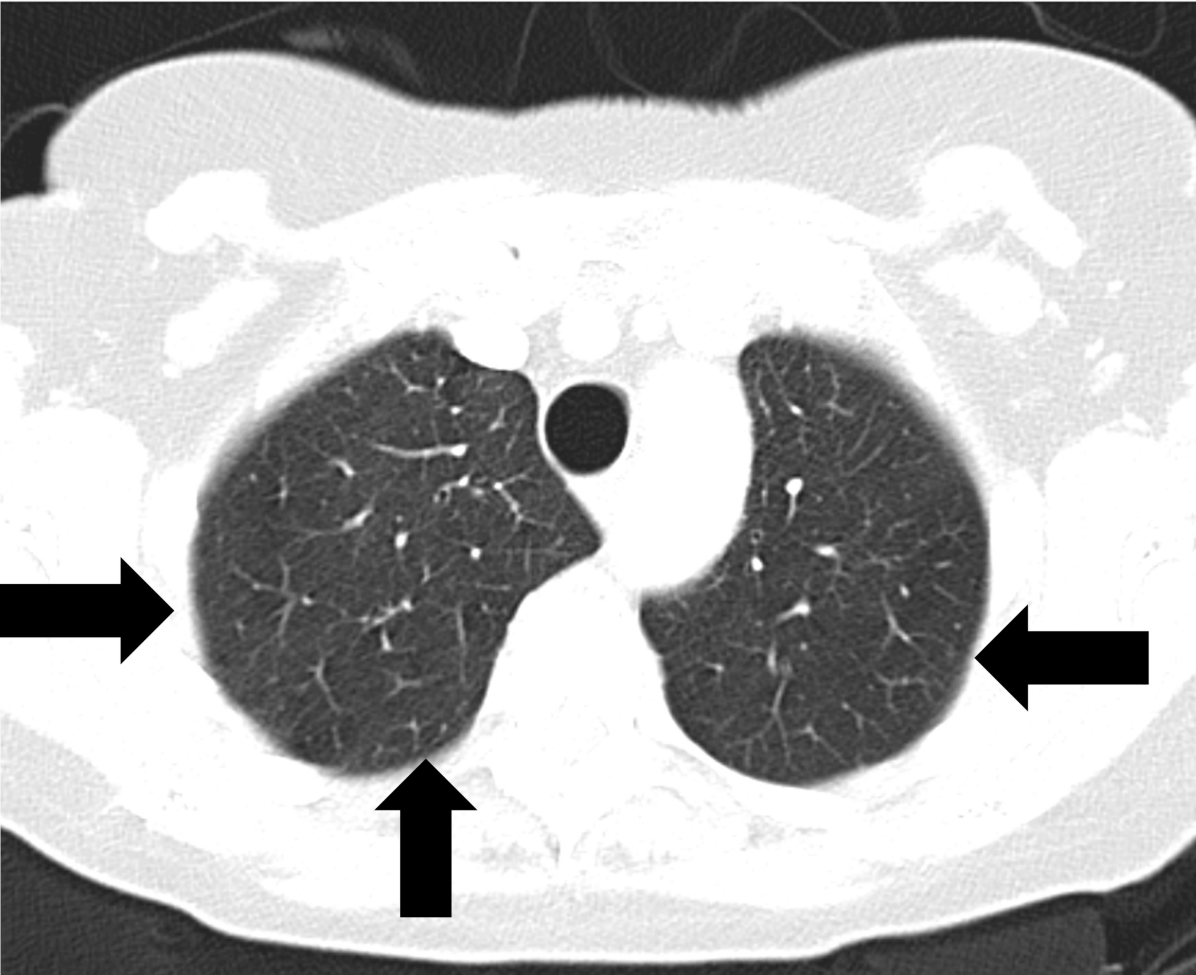
Thoracic CT scan showing the disappearance of all consolidation areas (second relapse). Absence of previously present parenchymal lesions (black arrows).

It was decided to reintroduce a high dose of corticosteroids (the same dosage of 30 mg/day as before) and re-evaluation after one month showed a radiological improvement and the patient was again asymptomatic (Figure 5).

After one month and a half, a new attempt on reducing the prednisolone dosage was made but it was followed once again by clinical deterioration. It was decided to initiate a long-term therapy with prednisolone 5 mg/day, erythromycin 250 mg three times a week, along with omeprazole 20 mg/day for three years. There have been no new relapses since then.

**Figure 6:**
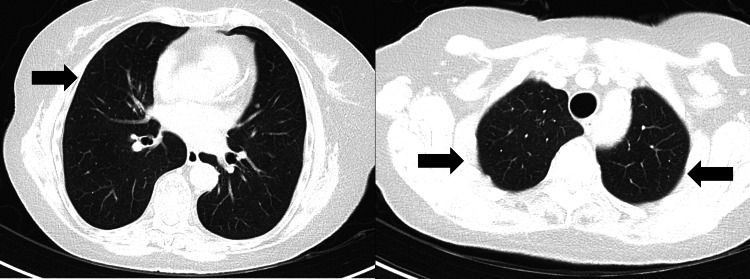
Thoracic CT scan 10 years after the first episode of radiation-induced organizing pneumonia. Presence of a few millimetric ground glass opacities (black arrows) surrounded by normal lung tissue.

A thoracic CT scan conducted at the 10-year mark showed only millimetric ground glass opacities (at the same level as the pulmonary infiltrates in Figure 4), with no inflammatory parenchymal or interstitial changes (Figure 6). To this day, the patient maintains a daily intake of prednisolone 5 mg/day, with no side effects.

At present, the patient has not experienced any new relapses and her oncological disease is stable.

## Discussion

OP, in both its cryptogenic and secondary form, is a rare interstitial lung disease having peculiar clinical, radiological, and pathological characteristics [7].

Since Epler reported OP (previously known as bronchiolitis obliterans organizing pneumonia) after postoperative irradiation for breast cancer in 1983, several reports described its peculiar features: subacute lung damage, which generally occurs within 12 months from the end of radiotherapy [8]. More recently, many advances have been made regarding OP's pathophysiology and possible risk factors and treatment (cryptogenic and secondary forms).

Cottin et al. presented a description of the pathophysiological mechanism of OP (cryptogenic or secondary) [9]: (1) an early phase of alveolar epithelial damage with desquamation of type I pneumocytes, which can form gaps in the basal lamina. In the alveolar lumen, the fibrin is deposited secondarily allowing the influx of inflammatory cells. (2) A proliferation phase, which is characterized by fibro-inflammatory buds. Fibrin is progressively fragmented by macrophages and inflammatory cells. The fibroblasts then migrate toward the area of damaged alveoli and gradually differentiate into myofibroblasts. (3) A last phase characterized by mature buds fibrotic in the alveolar space. Fibrin and inflammatory cells give way to organized myofibroblasts concentrically with collagen. In the remaining sequelae, the alveolar epithelium is gradually restored.

Ailloud et al. carried out a systematic review and concluded that the main risk factors are age, smoking, and the volume of lung irradiated [10].

King et al. presented a thorough description regarding treatment, emphasizing the main role played by glucocorticoid therapy as a first choice treatment and presenting a start dose of 0.5-1 mg/kg/day of prednisone, up to 60 mg per day, to be given as a single oral dose in the morning, for two to four weeks. It is proposed to taper the dose to 0.25 mg/kg/day to complete four to six months of therapy, followed by a gradual reduction to 0 mg over the next six to 12 months [11].

With regard to prognosis and response treatment, these appear to be generally excellent with no difference between the patients with or without relapse episodes [12].

We present a case from 2009 with a 16-year follow-up, in which the delay in prompt diagnosis and consequently incorrect treatment led to rapid deterioration of the patient.

Improvement of her clinical status varied between 10 days to one month from the introduction of high-dose corticosteroid therapy and was followed by tapering of corticosteroid therapy. Consequently, the patient developed three relapse episodes, all within a month/month and a half from the beginning of corticosteroid reduction. This highlights the challenge of the correct tapering of therapy in these cases, being it the dose rate or the period of time in which it must be made.

The third and last relapse episode was treated with a small daily dose of corticoid therapy (5 mg/day of prednisolone) coupled with a macrolide antibiotic (erythromycin 250 mg three times a week) for three years. Small, retrospective series have suggested that macrolide antibiotics with anti-inflammatory properties (e.g., erythromycin and clarithromycin) might be a useful adjunct to oral glucocorticoid therapy in patients with OP, with an administration time of three to six months or longer, with close monitoring during withdrawal of treatment [13,14].

This permitted a minimal dose of corticosteroids to be given to the patient, avoiding unwanted side effects, while allowing complete resolution and no new relapse episodes.

## Conclusions

The reported case emphasizes the diagnostic challenges and inherent complexity linked with OP secondary to radiation therapy. Particularly in the context of breast cancer, where adjuvant radiotherapy is a key component of conservative treatment, secondary OP emerges as a rare yet notable complication.

Corticosteroids are still the first line of treatment, with a few retrospective series showing a benefit in adding macrolide antibiotics, but the risk of relapses remains high requiring close management and continuous monitoring, which prompts the need for prospective, randomized treatment trials.
